# Reasons for reported suspicion of child maltreatment and responses from the child welfare - a cross-sectional study of Norwegian public dental health personnel

**DOI:** 10.1186/s12903-018-0490-x

**Published:** 2018-03-02

**Authors:** Ingfrid Vaksdal Brattabø, Ragnhild Bjørknes, Anne Nordrehaug Åstrøm

**Affiliations:** 1 Oral Health Centre of Expertise in Western Norway/Hordaland, Pb. 2354, Møllendal, 5867 Bergen, Norway; 2 Department of Health Promotion and Development, Faculty of Psychology, University of Bergen, Pb. 7807, 5020 Bergen, Norway; 3 Department of Clinical Dentistry, Faculty of Medicine and Dentistry, University of Bergen, Pb. 7804, 5020 Bergen, Norway

**Keywords:** Child maltreatment, Child abuse, Dental neglect, Sexual abuse, Oral health, Caries, Mandatory reporting, Child welfare services, Dental health services, Dentist

## Abstract

**Background:**

To prevent child maltreatment, the identification of vulnerable children is essential. In Norway, public dental health personnel (PDHP) report suspicion of child maltreatment to child welfare services (CWS) at a relatively high rate. However, their reasons for reporting and the response from CWS have not been investigated. The objectives of this study were to (1) explore the reasons that PDHP send reports of concern, (2) examine how CWS responds to PDHP reports, and (3) assess whether different reasons for concern are associated with a given response from CWS.

**Methods:**

A national cross-sectional study was conducted by an electronic survey distributed to public dental hygienists and dentists in Norway. Descriptive statistics were calculated in terms of mean (SD) distributions and frequency, expressed as % (n). To account for clustering of responses among respondents, binomial generalized estimating equation analysis was used to estimate odds ratios (ORs) and confidence intervals (CIs) of CWS responses across number of reports with different reasons for concern.

**Results:**

Of a total of 1542 questionnaire recipients, 1200 (77.8%) responded to the survey. From 2012 to 2014, 42.5% of the respondents sent 1214 reports to CWS, with a mean number of 2.7 (SD = 2.0) reports per respondent. The PDHP sent the reports due to suspicion of neglect or physical, sexual and/or psychological abuse. Non-attendance at dental appointments and grave caries were reported most frequently. Among the reports, 24.5% resulted in measures being taken by CWS, 20.7% were dropped, and 29.4% lacked information from CWS on the outcome. Reports due to suspicion of sexual abuse, (OR 1.979, 95% CI (1.047–3.742), *P* = 0.036), grave caries (OR 1.628, 95% CI (1.148–2.309), *P* = 0.006), and suspicion of neglect (OR 1.649, 95% CI (1.190–2.285), *P* = 0.003) had the highest association with the implementation of measures.

**Conclusions:**

PDHP report on several forms of child maltreatment and contributes in detection of victimized children. However, the relatively low number of measures being taken by CWS and the number of reports that lack a response to reporters reveal a need for a closer cooperation between the services, as this would benefit both the children at risk and the services.

**Electronic supplementary material:**

The online version of this article (10.1186/s12903-018-0490-x) contains supplementary material, which is available to authorized users.

## Background

### Child maltreatment

Being a victim of serious child maltreatment increases the risk of having developmental disturbances and reduces the possibility of having a normal and wholesome childhood. In fact, for many children, child maltreatment results in severe and lifelong challenges [1–3]. Child maltreatment is a widespread phenomenon worldwide [4]. There is a compelling body of research indicating that the maltreated children known to child welfare services (CWS) only represent the tip of the iceberg relative to the actual number of children being maltreated [4–7]. To prevent child maltreatment and its consequences, it is important to identify children at risk as well as children who are already victims. Such efforts require an interdisciplinary collaboration between the services working with children, and CWS is essential in this context.

### Mandatory reporting, the role of dental services and child welfare services

Several countries have enacted legislation mandating reporting of maltreatment, with the goal of increasing the reporting frequency among designated personnel working with children [8–10]. Research has indeed shown an increase in reporting frequency as reporting becomes mandatory [11, 12].

All health personnel in Norway are mandated by the Norwegian Health Personnel Act, section 6 § 33, to report suspicion of severe child maltreatment to CWS [13]. The Norwegian public dental health service (PDHS) is mandated by the Dental Health Service Act to prioritize the prevention of dental disease and offer all children under the age of 19 free and regular dental treatments [14]. As a result, close to 100% of all children in Norway are regularly covered by the PDHS. This situation gives the PDHS an important and unique opportunity to detect and report suspicion of child maltreatment to CWS. Public dental health personnel (PDHP) in Norway are experienced reporters of child maltreatment, with a total of 60% having reported suspicion of child maltreatment to CWS during their career [15].

The assignment of the Norwegian CWS reflects the general reporting legislation and the fact that Norway is a social-democratic welfare state [16]. The CWS is mandated by the Child Welfare Act, section 6 § 6-7a, to provide a response to reporters within three weeks, although a response is not mandatory if the concern is clearly unsubstantiated. In cases for which an investigation has been opened, CWS provides the reporter with information on whether the case has been dropped or measures have been taken [17].

### Previous research

Over the last decade, a number of studies have investigated the role of dental personnel in child maltreatment issues [15, 18–29]. High-quality research has focused on dental personnel, the frequency of reporting, failure to report, knowledge regarding child maltreatment and barriers to reporting [15, 18–20, 22, 24, 25, 28, 29]. However, little research has been focused on the reasons why dental personnel report to CWS [26, 30] and the associated responses from CWS. Additionally, to the best of our knowledge, no previous studies have explored how CWS responds to reports of concern from dental services and to what degree the Norwegian CWS fulfills the mandated response to designated reporters in this regard [17].

Although dental personnel’s barriers to and limited knowledge regarding reporting have been recognized and targeted in recent decades, the gap between suspecting child maltreatment and reporting seems to persist, implying that researchers have not succeeded in exploring all of the problems related to mandatory reporting [31]. To enhance our understanding and knowledge of the mechanisms involved in mandatory reporting in dental services, it is important to examine dental personnel’s reasons for sending a more thorough report of concern. Additionally, knowledge of how CWS responds to the different reports of concern sent by the PDHS should be obtained.

### Aims

Focusing on a census of public dentists and dental hygienists in Norway, the objectives of this study were threefold. First, we assessed the reasons reported by PDHP for having sent a report of concern to CWS during the three-year period from 2012 to 2014. Second, we examined how CWS responded to these reports. Third, we assessed whether the different reasons for sending a report of concern were associated with a given response from CWS.

## Methods

### Study design and data collection

This national cross-sectional study was conducted by an electronic survey in a census of dental health personnel employed by the PDHS in Norway. Specifically, an email explaining the purpose of the study and providing a link to the electronic questionnaire, also containing an informed consent form, were distributed to all dental hygienists and dentists. The chiefs of the PDHS provided the names and e-mail addresses of their employees and gave their employees permission to answer the survey questions during their working hours. The estimated time required to complete the questionnaire was 30–40 min.

The survey contained questions regarding experience with suspecting and reporting child maltreatment, reasons for reporting and/or not reporting, experience with CWS, organizational questions regarding PDHS and the demographic characteristics of the respondents. A portion of the questions was derived from an Australian survey [32, 33], with certain necessary adjustments to tailor the questions to dental personnel in a Norwegian context. In particular, the questions from the Australian questionnaire were translated into Norwegian and then back translated into English to evaluate the semantic and content equivalence. The questionnaire was reviewed by PDHP with experience in survey research and clinical work at the PDHS before it was piloted among PDHP in one county. After certain small adjustments, the questionnaire was distributed to the 18 remaining counties in Norway. The Ombudsman, Norwegian Social Science Data Services (NSD), approved and registered the survey and was responsible for distributing the questionnaire and collecting the data. The main survey was distributed in November 2014, and follow-ups with reminders were sent to non-responders after two, four and seven weeks. The questionnaire is available in Norwegian, as an additional file (see Additional file 1).

### Variables and their measurement

Experience with sending a report of concern to CWS was measured via the following question: ‘During your time as dental personnel, have you filed a report of concern due to suspicion of child abuse or neglect?’ The options were yes or no. Those answering yes were asked ‘Were any of the reports of concern sent in the time period from 2012 to this day?’ The response options were yes or no. If the respondent answered yes, they were asked ‘How many concerns have you filed since 2012?’ The response options ranged from one to ten or more concerns.

The respondents who had filed one or several reports of concern in the previous three years, from 2012 to 2014, were asked to provide the following information for each of the concerns that they reported having sent to CWS during this period: ‘The following questions regard the first report of concern that you sent in 2012–2014. What was the gender of the child in the first report of concern that you sent?’ The response options were boy or girl. ‘What was the age of the child in the first report of concern that you sent?’ The response options were 0–3, 4–7, 8–11, 12–15, and over 16 years. ‘What was the reason for the first report of concern that you sent? Multiple categories can be chosen.’ The response options were suspicion of physical abuse, suspicion of sexual abuse, suspicion of psychological abuse, suspicion of neglect, recurring missed appointment, grave caries, gingivitis, lack of hygiene, wounds and lesions in the oral cavity, trauma, other oral findings (please note), treatment refusal, cooperation with guardians, abnormal behavior, and other (please note). Wounds and lesions in the oral cavity and other oral findings were merged into one variable due to the low response frequency.

The background characteristics of the PDHP respondents that were assessed were gender, age (20–39 or 40+ years), occupation (dental hygienist or dentist), the number of patients treated in the last 12 months (0–500 or 501+ patients), the size of the municipality (0–10,000, 10,001–40,000, or 40,001+ inhabitants) and the geographical region where the dental clinic was located (north, central, west, south or east). More detailed information on the background characteristics can be found in a study by Brattabø et al., 2016 [15].

Regarding the responses from CWS, the PDHP were asked the following: ‘What response have you received from CWS regarding the first report of concern that you sent?’ The response options were as follows: ‘CWS has opened an investigation and taken measures’, ‘CWS has opened an investigation but dropped the case’, ‘CWS has opened an investigation but has not given me any feedback on whether measures have been taken or the case has been dropped’, ‘CWS has not opened an investigation, so the case has been dropped’, ‘CWS has not given any feedback’, ‘Other (please note)’, and ‘Do not know’.

The question battery described above regarding the reports of concern and corresponding responses from CWS was administered to each respondent repeatedly, the same number of times (1–10) that they had reported having sent a report of concern during the 2012–2014 period. Only the number of the report of concern mentioned in the questions was changed: ‘The following questions regard your [second, third, fourth, etc.] report of concern’.

### Statistical analysis

Data were analyzed using the Statistical Package for Social Sciences version 22 (SPSS Inc., Chicago, IL, USA). Descriptive statistics, in terms of frequency % (n) and mean (SD) distributions, were calculated. The frequency of the independent variables relative to the dependent variables was calculated using multiple responses, frequencies and cross tables. Due to the layout of the questionnaire and because each respondent could have sent up to ten reports of concern, variables were restructured from multiple variables to groups of related cases. Repeated data had a multilevel structure, with observations nested within individuals or clusters. To account for clustering in repeated data, responses from CWS were regressed on reasons for concern across the numbers of reports (first report of concern, second report of concern and so on) using the binomial generalized estimating equation (GEE) [34]. After restructuring the original data file from wide (number as a variable) to long (number as a case) configuration, the binomial logit function and exchangeable working correlation matrix were employed. CWS responses by reasons for concerns across number of reports were estimated using odds ratios (ORs) and 95% confidence intervals (CIs). Both unadjusted and adjusted GEE analyses were performed, and the significance level was set to *P* < 0.05.

For model building, a series of unadjusted and adjusted GEE models were fitted. Initial models were built by adding children’s age and gender and the PDHP’s specific occupation, number of patients treated, municipality size and geographical region in addition to the range of reasons for concern. As both the unadjusted and the adjusted analyses revealed no significant effect of gender, the age of the child, the number of patients treated, the size of the municipality or the geographical region, those variables were excluded from the final GEE model to strengthen the analysis. The final GEE model included occupation as the only background variable in addition to the range of reasons for concern, as mutually adjusted.

## Results

### Characteristics of the respondents

Of a total of 1542 questionnaire recipients, 1200 (77.8%) dentists and dental hygienists responded to the survey. As previously described [15], most of the 1200 respondents were women (80.3%) and dentists (68.9%), reflecting the present situation of the PDHS labor market in Norway [35]. The respondents had a mean of 11.9 (SD = 11.2) years of working experience, and 82.9% had examined more than 250 children under the age of 18 years during the previous 12 months. A total of 720 (60%) respondents had filed a report of concern during their career, with a mean of 3.6 (SD = 3.4) reports per experienced reporter, and 42.5% had filed a report during the three-year period from 2012 to 2014, with a mean number of 2.7 reports (SD = 2.0) per experienced reporter.

In the 2012–2014 period, the respondents reported having sent 1214 reports of concern to CWS, with 55.9% of the reports of concern regarding boys. The children had the following age distribution: 6.8% of the children were under the age of four, 35.6% were between 4 and 7 years, 31.6% were between 8 and 11 years, 20.5% were between 12 and 15 years, and 5.6% were between 16 and 17 years. Therefore, 74% of the children were under the age of 12 years.

### Reasons for concern

As shown in Table 1, the majority of the 2012–2014 reports of concern from PDHP were sent to CWS for multiple reasons, with a mean of 2.7 (SD = 1.8) reasons for concern per report. The most frequently reported reason for concern was ‘did not attend dental appointment’, which was cited in 67.4% of the reports. Grave caries was reported in nearly half of the reports of concern (49.2%), and lack of hygiene and suspicion of neglect were reported in 36.7% and 25.9% of the cases, respectively. Suspicion of physical abuse, sexual abuse and/or psychological abuse was cited in 4.9%, 4.7% and 4.4% of the reports, respectively.

**Table 1:** Reasons for sending reports of concern to CWS among PDHP in Norway, 1214 reports of concern, 3222 reasons for concern

Reason for sending a report of concern	*n*	% of reports
Did not attend/was not brought	818	67.4
Grave caries	597	49.2
Lack of hygiene	445	36.7
Suspicion of neglect	315	25.9
Interaction with parents/guardians	232	19.1
Abnormal behavior in the child	220	18.1
Treatment refusal	205	16.9
Gingivitis	119	9.8
Suspicion of physical abuse	59	4.9
Suspicion of sexual abuse	57	4.7
Suspicion of psychological abuse	53	4.4
Trauma	20	1.6
Wounds, lesions or other oral findings	14	1.2
Other	68	5.6

The frequencies do not sum to 100% due to multiple reasons for concern

### Frequency distribution of responses given by CWS to PDHP’s reports of concern

Different responses were provided by CWS to the reports of concern sent by the PDHP during the period from 2012 to 2014. Summing the first three columns depicted in Table 2, related to the cases in which CWS had opened an investigation and had taken measures, dropped the case or had not given any further information, revealed that 51.1% of the reports sent to CWS resulted in an investigation. Meanwhile, 4.6% of the reports were dropped without any investigation, and no feedback or information has been provided to the PDHP by CWS for 18.9% of the reports. Regarding the overall outcome from the PDHP reports of concern, 24.5% of reports of concern led to measures being taken by CWS, 20.7% of reports were dropped either after investigation or immediately, and CWS did not provide information on the outcome of the investigation or at all in 29.4% of reports. For the remaining 25.5% of reports, the dental personnel did not know or remember the outcome of their report of concern. Hence, the response estimates in Table 2 should be considered as minimum rates of occurrence.

**Table 2:** Frequency distribution of responses of CWS to reports of concern (1214) sent by PDHP in 2012–2014

Response from CWS	*n*	%
CWS has opened an investigation and taken measures	297	24.5
CWS has opened an investigation and dropped the case	195	16.1
CWS has opened an investigation, but no further information has been given	127	10.5
CWS has not opened an investigation, case dropped	56	4.6
CWS has not given any feedback at all	229	18.9
Do not know	310	25.5

### Reasons for sending report of concern and associated responses from CWS

Table 3 provides an overview of the numbers of times different reasons for concern were reported across various responses from CWS. The different reasons for concern led to initiatives by CWS with frequencies ranging from 19.1–40.4%. The reasons for concern that most frequently led CWS to open an investigation and take measures regarded suspicion of sexual abuse, trauma, suspicion of neglect and suspicion of physical abuse, with initiatives in response to 40.4%, 40.0%, 35.6% and 35.6% of the reports, respectively, in which reasons for concern were included. Furthermore, among a total of 818 reports of concern including the reason ‘did not attend’, only 21.4% resulted in initiatives by CWS; this reason for concern, together with wounds, lesions and other oral findings, led to the fewest initiatives by CWS. Reports of concern that included trauma or ‘did not attend’ were thus investigated and then dropped most frequently (accounting for 20.0% and 17.2% of cases, respectively).

**Table 3:** PDHP’s reason or multiple reasons for sending a report of concern with associated responses from CWS, 1214 reports 3222 reasons

	Response from CWS												
	CWS has opened an investigation and taken measures		CWS has opened an investigation and dropped the case		CWS has opened an investigation, but no further information has been given		CWS has not opened an investigation, case dropped		CWS has not given any feedback at all		Do not know		Total
PDHP’s reasons for sending a report	*n*	%	*n*	%	*n*	%	*n*	%	*n*	%	*n*	%	*n* total
Did not attend/was not brought	175	21.4	141	17.2	95	11.6	41	5.0	154	18.8	212	25.9	818
Grave caries	182	30.5	88	14.7	72	12.1	19	3.2	123	20.6	113	18.9	597
Lack of hygiene	136	30.6	64	14.4	55	12.4	16	3.6	95	21.3	79	17.8	445
Suspicion of neglect	112	35.6	45	14.3	39	12.4	9	2.9	65	20.6	45	14.3	315
Interaction with parents/guardians	69	29.7	34	14.7	33	14.2	8	3.4	48	20.7	40	17.2	232
Abnormal behavior in the child	69	31.4	23	10.5	36	16.4	8	3.6	45	20.5	39	17.7	220
Treatment refusal	56	27.3	34	16.6	27	13.2	7	3.4	42	20.5	39	19.0	205
Gingivitis	41	34.5	17	14.3	18	15.1	4	3.4	22	18.5	17	14.3	119
Suspicion of physical abuse	21	35.6	8	13.6	5	8.5	3	5.1	14	23.7	8	13.6	59
Suspicion of sexual abuse	23	40.4	9	15.8	6	10.5	0	0.0	9	15.8	10	17.5	57
Suspicion of psychological abuse	16	30.2	6	11.3	7	13.2	4	7.5	13	24.5	7	13.2	53
Trauma	8	40.0	4	20.0	4	20.0	0	0	3	15.0	1	5.0	20
Wounds, lesions or other oral findings	3	21.4	0	0	2	14.3	0	0	3	21.4	6	42.9	14
Other	13	19.1	7	10.3	7	10.3	0	0	16	23.5	25	36.8	68

### Responses from CWS according to PDHP’s reasons for sending report of concern

Table 4 depicts the results from adjusted GEE analyses, with each response from CWS regressed upon PDHP’s reasons of concerns across the number of reports. Each reason for concern was mutually adjusted for all other reasons for concern and for the PDHP’s specific occupation (a background factor). As shown, dental personnel sending reports of concern due to missed appointments were less likely to have their reports opened and substantiated by CWS than their counterparts sending reports without this reason (OR 0.667, 95% CI (0.469–0.949), *P* = 0.024). Dental personnel sending reports of concern that included suspicion of sexual abuse (OR 1.979, 95% CI (1.047–3.742), *P* = 0.036), grave caries (OR 1.628, 95% CI (1.148–2.309), *P* = 0.006), or suspicion of neglect (OR 1.649, 95% CI (1.190–2.285), *P* = 0.003) had a higher likelihood of having their reports opened and substantiated compared with dental personnel sending reports of concern without any of those reasons. Finally, reports of concern sent by dentists had a lower likelihood of being opened and substantiated compared with reports sent by dental hygienists (OR 0.623, 95% CI (0.425–0.916), *P* = 0.016).

**Table 4:** Generalized estimating equation analysis, with responses from CWS regressed upon PDHP’s reasons for reporting, adjusted analysis*

	CWS has opened an investigation and taken measures		CWS has opened an investigation and dropped the case		CWS has opened an investigation, but no further information has been given		CWS has not opened an investigation, case dropped		CWS has not given any feedback at all	
PDHP’s reason for reporting to CWS	OR (95% CI)	P	OR (95% CI)	P	OR (95% CI)	P	OR (95% CI)	P	OR (95% CI)	P
Did not attend/was not brought	0.667 (0.469–0.949)	.024	1.282 (0.829–1.980)	.264	1.522 (0.948–2.444)	.082	1.603 (0.822–3.125)	.166	1.323 (0.875–2.001)	.184
Grave caries	1.628 (1.148–2.309)	.006	0.904 (0.607–1.346)	.620	1.076 (0.675–1.713)	.759	0.684 (0.399–1.172)	.167	1.192 (0.838–1.697)	.329
Lack of hygiene	1.069 (0.755–1.514)	.707	0.834 (0.506–1.374)	.475	1.105 (0.643–1.899)	.717	0.702 (0.310–1.587)	.395	1.201 (0.812–1.775)	.359
Suspicion of neglect	1.649 (1.190–2.285)	.003	0.793 (0.499–1.259)	.326	0.967 (0.570–1.639)	.900	0.557 (0.259–1.197)	.134	1.249 (0.871–1.791)	.226
Interaction with parents/guardians	0.944 (0.648–1.377)	.767	1.092 (0.619–1.927)	.761	1.156 (0.674–1.985)	.598	1.079 (0.486–2.394)	.852	1.030 (0.687–1.545)	.885
Abnormal behavior in the child	1.392 (0.938–2.068)	.101	0.498 (0.284–0.847)	.015	1.779 (1.025–3.088)	.041	0.788 (0.220–2.819)	.714	0.838 (0.543–1.291)	.422
Treatment refusal	0.875 (0.587–1.305)	.513	1.270 (0.742–2.172)	.384	0.820 (0.507–1.327)	.420	1.134 (0.462–2.781)	.784	1.113 (0.746–1.661)	.599
Gingivitis	1.411 (0.908–2.192)	.126	0.955 (0.506–1.804)	.888	0.948 (0.430–2.091)	.895	0.780 (0.105–5.776)	.808	0.935 (0.569–1.537)	.792
Suspicion of physical abuse	1.352 (0.693–2.638)	.376	0.984 (0.403–2.402)	.972	0.687 (0.295–1.599)	.384	0.952 (0.278–3.264)	.938	1.601 (0.778–3.297)	.202
Suspicion of sexual abuse	1.979 (1.047–3.742)	.036	0.878 (0.327–2.359)	.797	0.755 (0.280–2.035)	.579	No numbers		0.870 (0.431–1.756)	.698
Suspicion of psychological abuse	0.783 (0.343–1.788)	.562	0.996 (0.384–2.580)	.993	1.188 (0.525–2.685)	.679	2.286 (0.777–6.730)	.133	1.211 (0.684–2.144)	.512
Trauma	1.613 (0.645–4.035)	.307	1.353 (0.347–5.271)	.663	2.358 (0.859–6.474)	.096	No numbers		0.507 (0.139–1.846)	.303
Wounds, lesions or other oral finds	0.831 (0.241–2.867)	.770	No numbers		1.659 (0.453–6.079)	.445	No numbers		1.669 (0.752–3.703)	.208
Dentist (dental hygienist is reference)	0.623 (0.425–0.916)	.016	0.866 (0.564–1.331)	.511	1.414 (0.807–2.476)	.226	0.870 (0.402–1.884)	.724	1.337 (0.855–2.092)	.203
Other	0.821 (0.388–1.739)	.606	0.975 (0.458–2.074)	.948	1.568 (0.683–3.854)	.327	No numbers		1.362 (0.603–3.074)	.458

* Mutually adjusted estimates; adjustments were made for PDHP’s reasons for reporting and PDHP’s specific occupation

Dental personnel sending reports of concern due to the abnormal behavior of the child were less likely to have their reports opened and then dropped (OR 0.498, 95% CI (0.284–0.847), *P* = 0.015), and more likely to have their reports opened without being given any further information by CWS (OR 1.779, 95% CI (1.025–3.088), *P* = 0.041) compared with their counterparts sending reports of concern without this reason.

## Discussion

The objectives of the present study were to explore PDHP’s reasons for sending a report of concern in the three-year period from 2012 to 2014, to assess how CWS responded to the reports of concern and to examine whether the different reasons for concern were associated with a given response from CWS. This study showed that Norwegian PDHP report on several types of suspected child maltreatment, including neglect and physical, psychological and sexual abuse. Thus, the majority of reports were sent due to multiple reasons for concern. Only one-fourth of the reports from the Norwegian PDHS led to a measure being taken and the PDHP lacked information regarding the outcome in approximately one third of the reports, while one-fifth were dropped either directly or after investigation. Reports due to suspicion of sexual abuse, grave caries and suspicion of neglect were most strongly associated with a response from the CWS in terms of having opened an investigation and implemented measures.

The most frequently reported reasons for concern were repeated failure to attend dental appointments, grave caries, a lack of hygiene and suspicion of neglect which is in accordance with findings in a Swedish study [30]. Repeated failure to dental attendance, could be attributed to forgetting, an address change, a lack of time, illness or dental anxiety [36–38]. This finding indicates also that PDHP and the PDHS are alerted when children continuously forfeit their legal right to free dental care according to the Public Dental Health Service Act [14]. In addition, when children repeatedly fail to attend their dental appointments, PDHP are placed in a position in which they are unable to fulfill their obligation to determine whether there is a need for dental treatment or oral health guidance. Previous studies have demonstrated associations of failure to attend a dental appointment, an absence of dental care routines, caries and poor dental health with families struggling with their everyday life and children having adverse childhood experiences [30, 36, 39–43]. This implies that that continuously missed dental appointments and dental neglect could be indicators of child maltreatment and could be used as a tool for the early identification of struggling children and families.

In the present study, children of all ages were reported to CWS, with close to three-quarters being under the age of 12 years. These findings indicate that dental personnel are in a position to detect children at risk especially those at a younger age. Concerning early detection of vulnerable children, this finding is of particular importance.

Regarding the reports of concern due to grave caries, it is important to be aware that recent statistics in Norway reveal that 82% of 5-year-olds and 60% of 12-year-olds had no experience with caries [35]. The good oral health of the majority of Norwegian children increases the conspicuousness of the children with extensive oral health problems. The present study suggests that PDHP are concerned for their patients with oral health deficiencies and suspect that these children may be neglected.

The results of the present study indicate competence and awareness among PDHP in Norway regarding the different forms of child maltreatment, even though potential cases of physical, psychological and sexual abuse were rarely reported. Increased focus during the recent years on child maltreatment-related issues in the PDHS, educational institutions, the media and among the authorities may be contributing factors in this regard. Present findings differ somewhat from findings in Sweden, where all the reports from dental service regarded concerns due to parental deficiencies (failure to attend appointments) and neglect (dental neglect), while concerns due to suspicion of psychological, sexual and/or physical abuse were absent [30]. However, the present findings are partly in accordance with findings from Greece, where dentists suspected several forms of child maltreatment, although they had very low reporting frequency [26]. In addition, studies from Denmark, the UK and Scotland have also shown that dental personnel reports child abuse and neglect, although without specifying what kind of child abuse and neglect is being reported [19, 20, 22]. The discrepancy with previous studies could be due to differences in sample size and study design. Small sample sizes reduces the chance of rare concerns being detected. Further, due to recall biases, social desirability, differences in definitions, reporting and registration there might be discrepancy between studies based upon self-reports and case-reports.

Only one-fourth of the reports from the Norwegian PDHS led to a measure being taken. Moreover, the PDHP lacked information regarding the outcome in approximately one third of the reports while one-fifth were dropped either directly or after investigation. This might be attributed to large workload of CWS as the numbers from Statistics Norway reveal a general increase in reports to CWS over the last few years [44]. Other plausible explanations might be overreporting or insufficient reports of concern from PDHP [45, 46]. One might further wonder if the frequency of measures being taken and the lack of information to PDHP is a result of unclear response procedures within CWS or lack of knowledge within CWS regarding dental neglect and its consequences. Specifically, in light of the good oral health in Norwegian children, it might be difficult for a CWS worker to fully understand the consequences that a lack of oral hygiene and treatment could have for a child. At present, however, this is only speculation, so additional research is needed.

According to the present findings, the odds of an investigation being opened and measures taken was 98% higher for reports of concern due to suspicion of sexual abuse compared with reports not due to this suspicion. Furthermore, suspicion of neglect and grave caries also showed increased odds of 65% and 63%, respectively for cases being opened and measures taken. The present findings suggest that CWS consider these concerns the most serious. In contrast, non-attendance at dental appointments seemed to be recognized as less serious reasons, with 33% lower odds of cases being opened and measures being taken compared with reports due to other suspicions. Hence, it may be reasonable to assume that CWS considers non-attendance more of an indication than a serious suspicion of child maltreatment. Meanwhile, reports including concern about abnormal behavior in the child had 50% lower odds of being dropped when first opened compared with reports without behavioral concerns, implying that CWS takes the behavior of children seriously. This study further show that, with the exception of abnormal behavior, no reason for concern was significantly associated with a case being dropped immediately or after investigation, which might indicate that CWS considers all types of reports from the PDHP.

The current findings might indicate that PDHP need to improve their reports of concern and clarify the severity of the consequences that a lack of oral hygiene and continuous missed appointments might have for a child. Furthermore, the present findings, with close to one third of the reports lacking information from CWS on the outcome, indicate that CWS should improve its feedback frequency to fulfill its obligation stemming from the Child Welfare Act. Overall, improvement of the cooperation and information flow between services will increase the knowledge of PDHP and CWS regarding the circumstances and needs of vulnerable children and will strengthen the wellbeing of these children.

For future research, there is a need to pinpoint whether continuously missed dental appointments and dental neglect are indicators of child maltreatment, serving as a tool for the early identification of struggling children and families. Furthermore, there is a need for research focusing on CWS and its experience with reports from and cooperation with PDHP. The present findings thus have implications for CWS, dental services, the authorities and future research.

Certain limitations of the present study should be noted. First, the findings mainly rely on self-reports of PDHP, which may undermine the study of the responses. Data were not collected from CWS, and hence, the perspective and experiences of CWS regarding reports of concern coming from PDHP and the response of CWS to reporters are not reflected. Second, the present study builds upon the experiences and recollections of PDHP regarding their contact with CWS during the three previous years. Therefore, there is a possibility of recall bias. In contrast, reporting to CWS is a challenging and rare event for most PDHP, likely increasing the likelihood of recall.

## Conclusion

This study shows that PDHP in Norway send reports to CWS regarding suspicion of the following different forms of child maltreatment: neglect and physical, sexual and/or psychological abuse. In general, PDHP reported that one-fourth of their reports of concern resulted in a measure being taken by CWS. Reports of concern regarding suspicion of sexual abuse, suspicion of neglect and/or grave caries had the highest likelihood of being opened and measures being taken, whereas non-attendance at dental appointments had the lowest likelihood.

The present findings indicate that dental personnel are in position to detect several forms of child maltreatment. However, the relatively low number of measures being taken by CWS and the number of reports that lack a response to reporters imply that closer and enhanced cooperation between CWS and PDHS is needed. This would benefit both the children at risk, the PDHS and the CWS in Norway.

## Additional file

Additional file 1: Tannhelse og barnevern - samhandling til beste for barnet. Questionnaire regarding dental personnel’s suspicion of child maltreatment and reporting to child welfare services. The questionnaire was sent to dental hygienists and dentists in the public dental health service in Norway 2014. (PDF 254 kb)

## Abbreviations

CWS: Child Welfare Service
GEE: Generalized Estimating Equations
NSD: Norwegian Social Science Data Services
PDHP: Public Dental Health Personnel
PDHS: Public Dental Health Service
